# Predicting the risk of gastroparesis in critically ill patients after CME using an interpretable machine learning algorithm – a 10-year multicenter retrospective study

**DOI:** 10.3389/fmed.2024.1467565

**Published:** 2025-01-06

**Authors:** Yuan Liu, Songyun Zhao, Wenyi Du, Wei Shen, Ning Zhou

**Affiliations:** ^1^Department of General Surgery, Wuxi People’s Hospital Affiliated to Nanjing Medical University, Wuxi, China; ^2^Department of Neurosurgery, Wuxi People’s Hospital Affiliated to Nanjing Medical University, Wuxi, China

**Keywords:** colonic neoplasms, intensive care unit, gastroparesis, prognosis, risk factor, machine learning

## Abstract

**Background:**

Gastroparesis following complete mesocolic excision (CME) can precipitate a cascade of severe complications, which may significantly hinder postoperative recovery and diminish the patient’s quality of life. In the present study, four advanced machine learning algorithms—Extreme Gradient Boosting (XGBoost), Random Forest (RF), Support Vector Machine (SVM), and *k*-nearest neighbor (KNN)—were employed to develop predictive models. The clinical data of critically ill patients transferred to the intensive care unit (ICU) post-CME were meticulously analyzed to identify key risk factors associated with the development of gastroparesis.

**Methods:**

We gathered 34 feature variables from a cohort of 1,097 colon cancer patients, including 87 individuals who developed gastroparesis post-surgery, across multiple hospitals, and applied a range of machine learning algorithms to construct the predictive model. To assess the model’s generalization performance, we employed 10-fold cross-validation, while the receiver operating characteristic (ROC) curve was utilized to evaluate its discriminative capacity. Additionally, calibration curves, decision curve analysis (DCA), and external validation were integrated to provide a comprehensive evaluation of the model’s clinical applicability and utility.

**Results:**

Among the four predictive models, the XGBoost algorithm demonstrated superior performance. As indicated by the ROC curve, XGBoost achieved an area under the curve (AUC) of 0.939 in the training set and 0.876 in the validation set, reflecting exceptional predictive accuracy. Notably, in the *k*-fold cross-validation, the XGBoost model exhibited robust consistency across all folds, underscoring its stability. The calibration curve further revealed a favorable concordance between the predicted probabilities and the actual outcomes of the XGBoost model. Additionally, the DCA highlighted that patients receiving intervention under the XGBoost model experienced significantly greater clinical benefit.

**Conclusion:**

The onset of postoperative gastroparesis in colon cancer patients remains an elusive challenge to entirely prevent. However, the prediction model developed in this study offers valuable assistance to clinicians in identifying key high-risk factors for gastroparesis, thereby enhancing the quality of life and survival outcomes for these patients.

## Introduction

Colon cancer is a malignant neoplasm that originates from the epithelial cells of the colonic mucosa, with a notably poor prognosis for affected patients. A significant proportion of these individuals require admission to the intensive care unit (ICU) for close monitoring and treatment due to complex clinical conditions or postoperative complications. Globally, approximately 8 million new cases of colon cancer are diagnosed annually, accounting for more than one-tenth of all newly diagnosed malignant tumors. As the modern diet, characterized by high fat, high meat, and low fiber intake, becomes increasingly prevalent, the incidence of colon cancer is expected to rise steadily (1). The current approach to treating colorectal cancer is primarily determined by the cancer’s stage, the patient’s overall health, and other individualized factors. However, radical surgical intervention remains the cornerstone of treatment. Hohenberger et al. (2) were the first to introduce complete mesocolic excision (CME), a surgical technique that involves the meticulous removal of both the tumor and the surrounding lymph nodes by excising the entire colonic mesentery and the associated lymphatic tissue in the region of the tumor. This procedure aims to achieve a higher tumor resection rate while minimizing the risk of recurrence (3). Pedrazzani et al.’s (4) retrospective study affirmed that the CME procedure ensures the complete excision of all cancerous tissue, thereby preventing the spread of the tumor to surrounding healthy tissues. Adhering to this principle has notably contributed to a significant reduction in local recurrence rates following surgery. Despite the notable benefits of CME in enhancing the outcomes and survival rates of colon cancer patients, the procedure is not without its risks, which can lead to complications that impact recovery and subsequent treatment. In some cases, these complications may necessitate admission to the ICU for further management. Gastroparesis, a condition characterized by impaired gastric emptying, is a frequently overlooked and often misdiagnosed complication following radical colon cancer surgery. While its incidence is more commonly associated with gastric cancer surgeries, its occurrence after colon cancer surgery should not be underestimated. Gastroparesis results from dysfunction in the nerves or muscles of the stomach, leading to delayed gastric emptying. Symptoms, including nausea, vomiting, bloating, and loss of appetite, often mimic the typical recovery process post-surgery. As such, these symptoms are frequently mistaken for normal postoperative reactions or mild dyspepsia, delaying or hindering timely diagnosis (5). Numerous studies have demonstrated that the development of gastroparesis in postoperative patients significantly heightens the risk of tumor recurrence and metastasis (6–8). Moreover, akin to other postoperative complications, gastroparesis leads to extended hospital stays and has increasingly become a formidable public health challenge worldwide (9). Gastroparesis, a prevalent complication following radical colon cancer surgery, not only imposes direct health risks but also has a profound impact on the financial wellbeing of patients and their families. Affected individuals may endure significant quality-of-life challenges, including malnutrition and diminished ability to perform daily activities, which can lead to a decrease in family income and an increased financial burden. Consequently, accurately predicting the onset of gastroparesis following total mesocolic excision and identifying high-risk patients is of paramount importance.

Surgeons typically assess the risk of gastroparesis in surgical patients based on clinical experience and examination reports; however, this approach has its limitations. On one hand, surgeons often rely on their own professional judgment and clinical expertise, leading to varying assessments of the same condition, which can be somewhat subjective. In more complex or rare cases, exclusive reliance on experience may result in biased evaluations. On the other hand, while clinical examinations and laboratory tests (such as blood tests and gastric emptying scans) provide valuable supporting information, they typically reflect the patient’s current status and may not offer an accurate prediction of postoperative gastroparesis risk. Some clinicians also employ traditional linear models and logistic regression for risk factor studies of postoperative gastroparesis in an effort to improve prediction accuracy (10). However, the development of postoperative gastroparesis is rarely attributable to a single factor; rather, it results from the interplay of multiple factors, such as the type of surgery, patient age, underlying comorbidities, intraoperative manipulations, and anesthesia techniques. Traditional regression models typically assume the independence of variables, disregarding the complex interactions among these factors. This limitation has prompted clinical researchers to acknowledge that regression models alone are insufficient for addressing challenges in clinical disease prediction. In recent years, with the rapid advancements in data science and machine learning, an increasing number of studies have shifted toward more sophisticated algorithms, such as random forests, support vector machines, and deep learning (11). These machine learning techniques excel at discerning the unique characteristics of different patient types within vast datasets, thereby facilitating the development of personalized medical solutions. Each patient’s condition, genetic makeup, lifestyle, and other factors are distinct, yet traditional medical treatments often adhere to a “one-size-fits-all” approach. In contrast, machine learning can analyze detailed patient data to identify specific treatment needs, enabling more tailored and effective healthcare strategies (12).

In this study, a machine learning model was developed to predict high-risk factors for gastroparesis following CME for colon cancer, by analyzing the clinical data of critically ill patients in ICU wards. This model is capable of identifying high-risk individuals at risk of developing gastroparesis after colon cancer CME, without the need for conventional imaging techniques, such as abdominal CT, thereby offering a potential means to reduce healthcare costs.

## Materials and methods

### Study subjects

In this study, we utilized clinical data from two medical institutions: Wuxi People’s Hospital, affiliated with Nanjing Medical University, and Wuxi Second People’s Hospital. The inclusion criteria for cases were as follows: (1) patients who underwent laparoscopic-assisted CME or traditional open CME; (2) all patients were transferred to the ICU due to postoperative complications; (3) the surgical team consisted of senior physicians skilled in independently performing CME; and (4) postoperative pathology confirmed a diagnosis of colorectal cancer. Exclusion criteria included: (1) patients with concurrent malignant tumors; (2) patients with distant metastasis of colon cancer confirmed through pathological examination or imaging; (3) patients with severe cardiovascular or respiratory conditions; (4) patients with significant organ dysfunction, such as liver or kidney disease; and (5) patients with incomplete clinical data, missing cases, or lost to follow-up. All patients were followed for a minimum of 3 years post-surgery. This retrospective study was approved by the Ethics Committees of Wuxi People’s Hospital and Wuxi Second People’s Hospital, and was conducted with patient consent, with all personal information anonymized. The ethical approval number for this study is KY22086.

### Study design and data collection

The dataset included 34 preoperative variables (collected within 24 h before surgery), intraoperative variables, and postoperative variables (assessed 48 h after the initial surgery). Preoperative variables encompassed patient demographics (gender, age, smoking history, alcohol use, and body mass index), fundamental clinical characteristics (American Society of Anesthesiologists score, Nutrition Risk Screening 2002 score, history of prior surgeries, adjuvant chemotherapy, and adjuvant radiotherapy), medical history (anemia, diabetes, hypothyroidism, hypertension, chronic obstructive pulmonary disease, hyperlipidemia, and coronary artery disease), laboratory test results (albumin, carcinoembryonic antigen, and carbohydrate antigen 19-9), and tumor characteristics (T-stage, N-stage, peripheral nerve invasion, tumor size, and tumor number). Intraoperative variables included the type of surgery, surgical approach, surgery duration, intraoperative blood loss, blood transfusions, and percutaneous arterial oxygen saturation levels. Postoperative variables consisted of laboratory indices (procalcitonin, C-reactive protein, and serum amyloid A). The primary outcome of this study was the incidence of postoperative gastroparesis.

### Diagnosis of gastroparesis

The diagnostic criteria for postoperative gastroparesis are as follows: (1) the presence of gastrointestinal symptoms, including nausea, vomiting, early satiety, bloating, or epigastric pain; (2) the exclusion of other conditions that may present with similar gastrointestinal symptoms, such as mechanical obstruction, drug-induced side effects, or metabolic disorders; (3) the elimination of confounding factors, such as the use of medications that may impair smooth muscle contraction; and (4) the confirmation of delayed gastric emptying through transgastric scintigraphy or magnetic resonance imaging (13, 14).

### Development and evaluation of predictive models for machine learning algorithms

In the present study, statistical analyses were performed using SPSS and R software. The construction and evaluation of the clinical prediction models involved the following steps: (1) Data preprocessing: colon cancer patients from Wuxi People’s Hospital between January 2010 and January 2020 were designated as the internal validation set, while patients from Wuxi Second People’s Hospital during the same period served as the external validation set. The internal validation set was randomly divided into a training set (70%) and a test set (30%). This approach strikes a balance between evaluating model performance and generalization ability, allowing the model to be trained on a substantial portion of the data while reserving a portion for testing the model’s predictive accuracy. Furthermore, given the moderate size of the dataset, this ratio ensures the training set contains a sufficient number of samples to capture key patterns and features, while the 30% test set provides an adequate sample for validating the model’s generalizability. (2) Univariate and multivariate regression analyses were performed on the internal validation set data. The Chi-square test was applied to categorical variables, while the *t*-test was used for continuous variables with a normal distribution. For continuous variables that were not normally distributed, the rank sum test was employed. A *p*-value of less than 0.05 was considered statistically significant. Logistic regression analysis was conducted on variables identified as significant in univariate analysis to assess their independent effects on postoperative gastroparesis. Four models—Extreme Gradient Boosting (XGBoost), Random Forest (RF), Support Vector Machine (SVM), and *k*-nearest neighbor (KNN)—were utilized to evaluate the importance of each factor and rank them accordingly. Variables that ranked in the top 10 across all 4 models and were deemed meaningful in both univariate and multivariate analyses were selected. These four models represent different types of machine learning algorithms: tree-based models (XGBoost and RF), KNN, and SVM. By combining these diverse model types, the limitations of any single algorithm can be mitigated, providing a more comprehensive and objective evaluation of factor importance. Both XGBoost and RF are integrated decision-tree-based models that inherently produce feature importance scores. These models are well-suited to handle non-linear relationships and complex interactions, making them highly effective for analyzing datasets with numerous variables and identifying key factors. SVM, with its strong generalization capability, is particularly suited to situations with small sample sizes, allowing the model to remain sensitive to a few key variables while minimizing the risk of overfitting. KNN, despite being sensitive to data noise, offers valuable insights in small sample or local similarity analyses, providing an intuitive reflection of the relationship between variables and outcomes. By comparing these different algorithms, a more holistic assessment of each factor’s importance can be made, and key variables that perform consistently well across multiple models can be identified, ensuring that the selected important factors possess greater applicability and robustness under varied prediction conditions. (3) Evaluate and build prediction models: the refined feature variables were used as input labels for the four machine learning algorithms—SVM, RF, XGBoost, and KNN. Differentiation, calibration, and clinical utility are key evaluation criteria for assessing predictive models. Each criterion highlights a distinct aspect of model performance and provides a comprehensive assessment of the model’s quality and practical value. Discrimination measures the ability of a model to distinguish between positive and negative samples (e.g., diseased versus undiseased). A high discriminative power indicates that the model can effectively differentiate between distinct categories of individuals. The area under the curve (AUC) value was derived from plotting receiver operating characteristic (ROC) curves to evaluate the model’s performance across various thresholds. The closer the AUC is to 1, the better the model’s discriminatory ability. Calibration assesses the agreement between the predicted probabilities and the actual outcomes, reflecting the model’s “reliability.” A well-calibrated model precisely predicts the actual occurrence rate for a given probability, ensuring that the model’s output aligns closely with real-world observations. We plotted calibration curves, grouping the probabilities predicted by the model and comparing them to the actual rates of occurrence. Ideally, the calibration curve should follow a 45 diagonal, representing perfect calibration. Deviations from this diagonal indicate discrepancies between the predicted probabilities and actual outcomes, which may manifest as overestimations or underestimations. Clinical utility assesses the real-world value of a model in clinical decision-making, specifically the benefit it brings to both patients and healthcare providers. It focuses on how a predictive model influences patient health outcomes across various thresholds. To analyze clinical utility, we used decision curve analysis (DCA), which evaluates the net benefit at different prediction thresholds. Net Benefit (NB) is calculated as the benefit derived from positive model predictions minus the cost of misclassification at a given threshold. DCA helps determine whether the model offers substantial clinical value at specific thresholds. Internal validation was conducted using *k*-fold cross-validation. In this method, the dataset is randomly divided into *k* subsets (or folds) of approximately equal size. Typically, *k* is set to 10 (i.e., 10-fold cross-validation), although this value can be adjusted based on dataset size and specific needs. Ten-fold cross-validation is commonly used as it strikes a balance between bias and variance while optimizing computational efficiency and model stability. In each iteration, one subset serves as the test set, while the remaining *k*-1 subsets are used for training. This process allows the model to be constructed and evaluated *k* times, with each subset serving as the test set once. Evaluation metrics such as AUC, accuracy, sensitivity, and specificity are recorded in each iteration. The final model performance is the average result of these *k* iterations, providing a comprehensive assessment of model stability across different data divisions. (4) External validation of the best model: the generalizability and predictive efficiency of the optimal model were assessed by applying it to an external validation set from an independent cohort. The model’s performance was again evaluated using ROC curves to confirm its robustness and ability to accurately predict postoperative gastroparesis in patients outside the original training dataset. This step ensures that the model’s performance is not limited to the internal dataset and that it can be effectively used in real-world clinical settings. (5) Model interpretation: to interpret the model’s predictions and gain insight into the role of different features, Shapley Additive Explanations (SHAP) were employed. SHAP values provide a clear explanation of how each feature contributes to the model’s predictions. The SHAP summary plot visualizes the importance of each feature, ranking them based on their impact on the model’s decision. For individualized patient predictions, the SHAP force plot was used, which demonstrates the influence of each feature on the predicted risk of gastroparesis. The SHAP force diagram calculates and displays the contribution of each feature to the predicted value, showing which variables increase or decrease the likelihood of gastroparesis for an individual patient. This allows clinicians to pinpoint the key risk factors specific to each patient and make more informed, personalized clinical decisions.

## Results

### Basic clinical information of the patient

**FIGURE 1:**
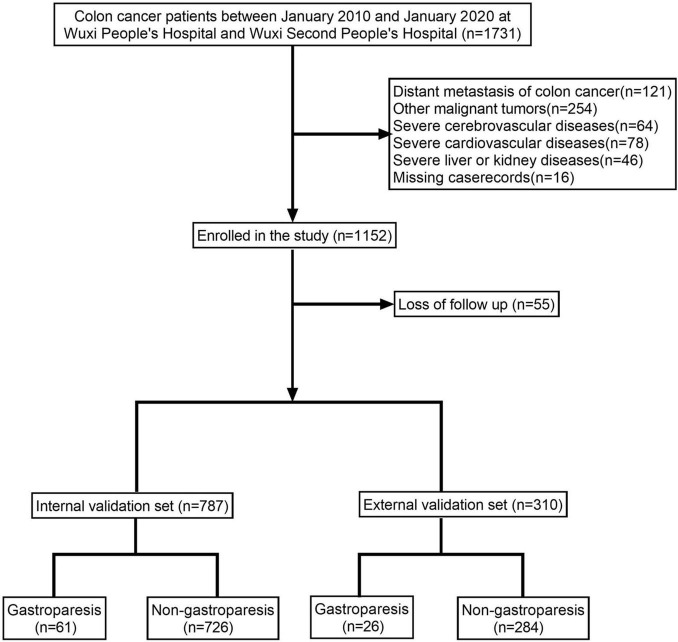
Flow diagram of patients included in the study.

The study encompassed a total of 1,097 colon cancer patients, of which 87 patients (7.93%) were diagnosed with gastroparesis (Figure 1 and Table 1). The internal validation set consisted of 787 colon cancer patients, with 61 patients (7.75%) diagnosed with gastroparesis. The external validation set included 310 colon cancer patients, of whom 26 patients (8.39%) had gastroparesis. The comprehensive original dataset underpinning this study is provided in Supplementary Table 1. The code utilized in this research has been uploaded to NutCloud, accessible via the following link: https://www.jianguoyun.com/p/DWh9chMQl-GKDBjEj-sFIAA.

**TABLE 1:** Preoperation and intraoperative information.

Variables		All (*n* = 1,146)	Non-gastroparesis (*n* = 1,051)	Gastroparesis (*n* = 95)	*p*-Value
Sex	Female	362 (45.997)	331 (45.592)	31 (50.820)	0.431
	Male	425 (54.003)	395 (54.408)	30 (49.180)	
Age	65	604 (76.747)	576 (79.339)	28 (45.902)	<0.001
	≥65	183 (23.253)	150 (20.661)	33 (54.098)	
BMI	25 kg/m^2^	521 (66.201)	488 (67.218)	33 (54.098)	0.037
	≥25 kg/m^2^	266 (33.799)	238 (32.782)	28 (45.902)	
ASA	3	512 (65.057)	477 (65.702)	35 (57.377)	0.19
	≥3	275 (34.943)	249 (34.298)	26 (42.623)	
Drinking history	No	538 (68.361)	495 (68.182)	43 (70.492)	0.709
	Yes	249 (31.639)	231 (31.818)	18 (29.508)	
Smoking history	No	541 (68.742)	507 (69.835)	34 (55.738)	0.023
	Yes	246 (31.258)	219 (30.165)	27 (44.262)	
ALB	≥30 g/L	568 (72.173)	543 (74.793)	25 (40.984)	<0.001
	30 g/L	219 (27.827)	183 (25.207)	36 (59.016)	
NRS2002 score	3	543 (68.996)	499 (68.733)	44 (72.131)	0.582
	≥3	244 (31.004)	227 (31.267)	17 (27.869)	
Surgical history	No	483 (61.372)	459 (63.223)	24 (39.344)	<0.001
	Yes	304 (38.628)	267 (36.777)	37 (60.656)	
Anemia	No	581 (73.825)	553 (76.171)	28 (45.902)	<0.001
	Yes	206 (26.175)	173 (23.829)	33 (54.098)	
Hyperlipidemia	No	641 (81.449)	597 (82.231)	44 (72.131)	0.051
	Yes	146 (18.551)	129 (17.769)	17 (27.869)	
Hypertension	No	413 (52.478)	389 (53.581)	24 (39.344)	0.032
	Yes	374 (47.522)	337 (46.419)	37 (60.656)	
Diabetes	No	638 (81.067)	613 (84.435)	25 (40.984)	<0.001
	Yes	149 (18.933)	113 (15.565)	36 (59.016)	
Hypothyroidism	No	585 (74.333)	558 (76.860)	27 (44.262)	<0.001
	Yes	202 (25.667)	168 (23.140)	34 (55.738)	
COPD	No	651 (82.719)	607 (83.609)	44 (72.131)	0.023
	Yes	136 (17.281)	119 (16.391)	17 (27.869)	
CHD	No	681 (86.531)	629 (86.639)	52 (85.246)	0.76
	Yes	106 (13.469)	97 (13.361)	9 (14.754)	
Adjuvant radiotherapy	No	597 (75.858)	559 (76.997)	38 (62.295)	0.01
	Yes	190 (24.142)	167 (23.003)	23 (37.705)	
Adjuvant chemotherapy	No	570 (72.427)	531 (73.140)	39 (63.934)	0.122
	Yes	217 (27.573)	195 (26.860)	22 (36.066)	
Surgical procedure	Laparoscopic surgery	630 (80.051)	591 (81.405)	39 (63.934)	0.001
	Open surgery	157 (19.949)	135 (18.595)	22 (36.066)	
Emergency surgery	No	576 (73.189)	535 (73.691)	41 (67.213)	0.273
	Yes	211 (26.811)	191 (26.309)	20 (32.787)	
Surgery time	270 min	498 (63.278)	470 (64.738)	28 (45.902)	0.003
	≥270 min	289 (36.722)	256 (35.262)	33 (54.098)	
Intraoperative bleeding	100 ml	527 (66.963)	504 (69.421)	23 (37.705)	<0.001
	≥100 ml	260 (33.037)	222 (30.579)	38 (62.295)	
Blood transfusion	No	637 (80.940)	589 (81.129)	48 (78.689)	0.641
	Yes	150 (19.060)	137 (18.871)	13 (21.311)	
SPO_2_	≥90%	633 (80.432)	584 (80.441)	49 (80.328)	0.983
	90%	154 (19.568)	142 (19.559)	12 (19.672)	
T-stage	T1∼T2	559 (71.029)	530 (73.003)	29 (47.541)	<0.001
	T3∼T4	228 (28.971)	196 (26.997)	32 (52.459)	
N-stage	N0	562 (71.410)	528 (72.727)	34 (55.738)	0.005
	N1∼N2	225 (28.590)	198 (27.273)	27 (44.262)	
PNI	No	705 (89.581)	654 (90.083)	51 (83.607)	0.112
	Yes	82 (10.419)	72 (9.917)	10 (16.393)	
Tumor number	2	595 (75.604)	570 (78.512)	25 (40.984)	<0.001
	≥2	192 (24.396)	156 (21.488)	36 (59.016)	
Tumor size	5 cm	536 (68.107)	510 (70.248)	26 (42.623)	<0.001
	≥5 cm	251 (31.893)	216 (29.752)	35 (57.377)	
CEA level	5 ng/ml	575 (73.062)	524 (72.176)	51 (83.607)	0.053
	≥5 ng/ml	212 (26.938)	202 (27.824)	10 (16.393)	
CA199 level	37 U/mL	583 (74.079)	539 (74.242)	44 (72.131)	0.718
	≥37 U/mL	204 (25.921)	187 (25.758)	17 (27.869)	
PCT level	0.05 ng/ml	571 (72.554)	526 (72.452)	45 (73.770)	0.825
	≥0.05 ng/ml	216 (27.446)	200 (27.548)	16 (26.230)	
CRP level	10 mg/L	530 (67.344)	497 (68.457)	33 (54.098)	0.022
	≥10 mg/L	257 (32.656)	229 (31.543)	28 (45.902)	
SAA level	10 mg/L	557 (70.775)	521 (71.763)	36 (59.016)	0.036
	≥10 mg/L	230 (29.225)	205 (28.237)	25 (40.984)	

OR, odds ratio; CI, confidence interval; BMI, body mass index; ASA, The American Society of Anesthesiologists; ALB, albumin; CA125, carbohydrate antigen 125; CA19-9, carbohydrate antigen 19-9; PCT, procalcitonin; CRP, C-reactive protein; SAA, serum amyloid A; NRS2002, nutrition risk screening 2002; CHD, coronary heart disease; COPD, chronic obstructive pulmonary disease; SPO_2_, percutaneous arterial oxygen saturation.

### Screening for risk factors for postoperative gastroparesis

**FIGURE 2:**
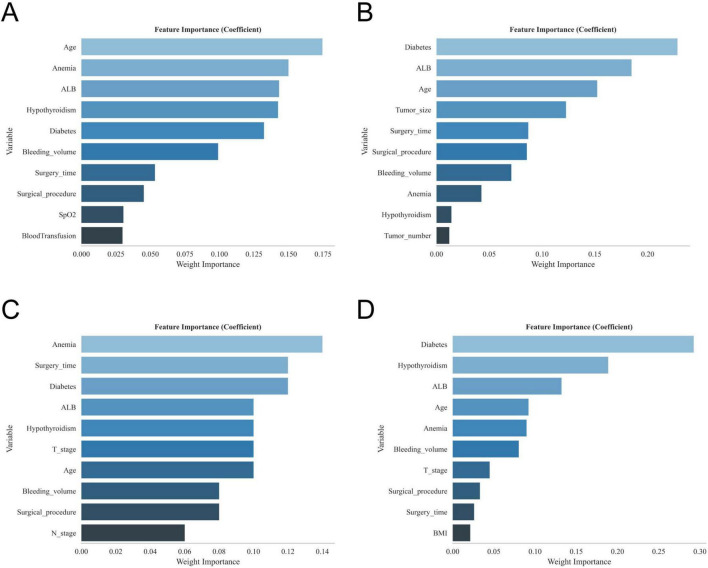
The variable ranking plots of the four models. **(A)** Variable importance ranking diagram of the XGBoost model. **(B)** Variable importance ranking diagram of the RF model. **(C)** Variable importance ranking diagram of the SVM model. **(D)** Variable importance ranking diagram of the KNN model.

The results of univariate and multivariate analyses identified several independent factors influencing the occurrence of postoperative gastroparesis, including age, albumin (ALB) levels, history of anemia, history of diabetes mellitus, history of hypothyroidism, history of adjuvant radiotherapy, type of surgery, duration of surgery, intraoperative bleeding, tumor size, and number of tumors (*p* < 0.05) (Table 2). The XGBoost, RF, SVM, and KNN models further identified key risk factors for postoperative gastroparesis, which included advanced age, hypoproteinemia, history of anemia, history of diabetes mellitus, history of hypothyroidism, open surgery, long operative time, and high intraoperative bleeding (Figures 2A–D). Based on a comprehensive analysis of these factors, the prediction model incorporated the following variables: age ≥65, hypoproteinemia, history of anemia, history of diabetes mellitus, history of hypothyroidism, open surgery, operative time ≥270 min, and intraoperative bleeding ≥100 ml.

**TABLE 2:** Univariate and multivariate analysis of variables related to gastroparesis.

Variables		Univariate analysis		Multivariate analysis	
		**OR, 95% CI**	***p*-Value**	**OR, 95% CI**	***p*-Value**
Sex	Female	Reference			
	Male	0.81 [0.48, 1.37]	0.432		
Age	65	Reference		Reference	
	≥65	4.53 [2.65, 7.72]	<0.001	3.76 [1.73, 8.21]	<0.001
BMI	25 kg/m^2^	Reference		Reference	
	≥25 kg/m^2^	1.74 [1.03, 2.95]	0.039	1.34 [0.64, 2.83]	0.441
ASA	3	Reference			
	≥3	1.42 [0.84, 2.42]	0.192		
Drinking history	No	Reference			
	Yes	0.90 [0.51, 1.59]	0.71		
Smoking history	No	Reference		Reference	
	Yes	1.84 [1.08, 3.12]	0.024	1.10 [0.51, 2.38]	0.809
ALB	≥30 g/L	Reference		Reference	
	30 g/L	4.27 [2.50, 7.31]	<0.001	2.81 [1.30, 6.06]	0.009
NRS2002 score	3	Reference			
	≥3	0.85 [0.47, 1.52]	0.582		
Surgical history	No	Reference		Reference	
	Yes	2.65 [1.55, 4.53]	<0.001	1.47 [0.69, 3.14]	0.32
Anemia	No	Reference		Reference	
	Yes	3.77 [2.21, 6.41]	<0.001	3.67 [1.71, 7.89]	<0.001
Hyperlipidemia	No	Reference			
	Yes	1.79 [0.99, 3.23]	0.054		
Hypertension	No	Reference		Reference	
	Yes	1.78 [1.04, 3.04]	0.034	1.35 [0.63, 2.89]	0.442
Diabetes	No	Reference		Reference	
	Yes	7.81 [4.51, 13.52]	<0.001	5.12 [2.38, 11.02]	<0.001
Hypothyroidism	No	Reference		Reference	
	Yes	4.18 [2.45, 7.13]	<0.001	3.94 [1.85, 8.39]	<0.001
COPD	No	Reference		Reference	
	Yes	1.97 [1.09, 3.57]	0.025	2.19 [0.91, 5.27]	0.08
CHD	No	Reference			
	Yes	1.12 [0.54, 2.35]	0.76		
Adjuvant radiotherapy	No	Reference		Reference	
	Yes	2.03 [1.17, 3.50]	0.011	2.40 [1.06, 5.42]	0.036
Adjuvant chemotherapy	No	Reference			
	Yes	1.54 [0.89, 2.66]	0.125		
Surgical procedure	Laparoscopic surgery	Reference		Reference	
	Open surgery	2.47 [1.42, 4.30]	0.001	3.24 [1.45, 7.25]	0.004
Emergency surgery	No	Reference			
	Yes	1.37 [0.78, 2.39]	0.274		
Surgery time	270 min	Reference		Reference	
	≥270 min	2.16 [1.28, 3.66]	0.004	2.30 [1.08, 4.90]	0.032
Intraoperative bleeding	100 ml	Reference		Reference	
	≥100 ml	3.75 [2.18, 6.44]	<0.001	3.46 [1.61, 7.40]	0.001
Blood transfusion	No	Reference			
	Yes	1.16 [0.61, 2.21]	0.641		
SPO_2_	≥90%	Reference			
	90%	1.01 [0.52, 1.94]	0.983		
T-stage	T1∼T2	Reference		Reference	
	T3∼T4	2.98 [1.76, 5.06]	<0.001	1.39 [0.65, 2.98]	0.393
N-stage	N0	Reference		Reference	
	N1∼N2	2.12 [1.25, 3.60]	0.006	1.89 [0.88, 4.03]	0.101
PNI	No	Reference			
	Yes	1.78 [0.87, 3.66]	0.116		
Tumor number	2	Reference		Reference	
	≥2	5.26 [3.07, 9.03]	<0.001	3.65 [1.76, 7.58]	<0.001
Tumor size	5 cm	Reference		Reference	
	≥5 cm	3.18 [1.87, 5.41]	<0.001	3.67 [1.74, 7.77]	<0.001
CEA level	5 ng/ml	Reference			
	≥5 ng/ml	0.51 [0.25, 1.02]	0.057		
CA199 level	37 U/ml	Reference			
	≥37 U/ml	1.11 [0.62, 2.00]	0.718		
PCT level	0.05 ng/ml	Reference			
	≥0.05 ng/ml	0.94 [0.52, 1.69]	0.825		
CRP level	10 mg/L	Reference		Reference	
	≥10 mg/L	1.84 [1.09, 3.12]	0.023	1.43 [0.66, 3.09]	0.365
SAA level	10 mg/L	Reference		Reference	
	≥10 mg/L	1.76 [1.03, 3.01]	0.037	1.00 [0.45, 2.19]	0.995

OR, odds ratio; CI, confidence interval; BMI, body mass index; ASA, The American Society of Anesthesiologists; ALB, albumin; CA125, carbohydrate antigen 125; CA19-9, carbohydrate antigen 19-9; PCT, procalcitonin; CRP, C-reactive protein; SAA, serum amyloid A; NRS2002, nutrition risk screening 2002; CHD, coronary heart disease; COPD, chronic obstructive pulmonary disease; SPO_2_, percutaneous arterial oxygen saturation.

### Model building and evaluation

**FIGURE 3:**
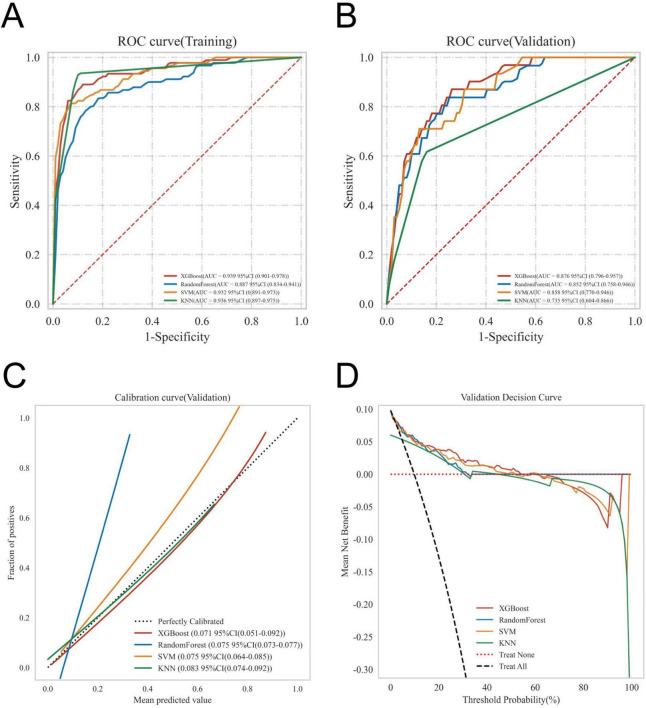
Evaluation of the four models for predicting gastroparesis. **(A)** ROC curves for the training set of the four models. **(B)** ROC curves for the validation set of the four models. **(C)** Calibration plots of the four models. The 45° dotted line on each graph represents the perfect match between the observed (*y*-axis) and predicted (*x*-axis) complication probabilities. A closer distance between two curves indicates greater accuracy. **(D)** DCA curves of the four models. The intersection of the red curve and the All curve is the starting point, and the intersection of the red curve and the None curve is the node within which the corresponding patients can benefit.

**FIGURE 4:**
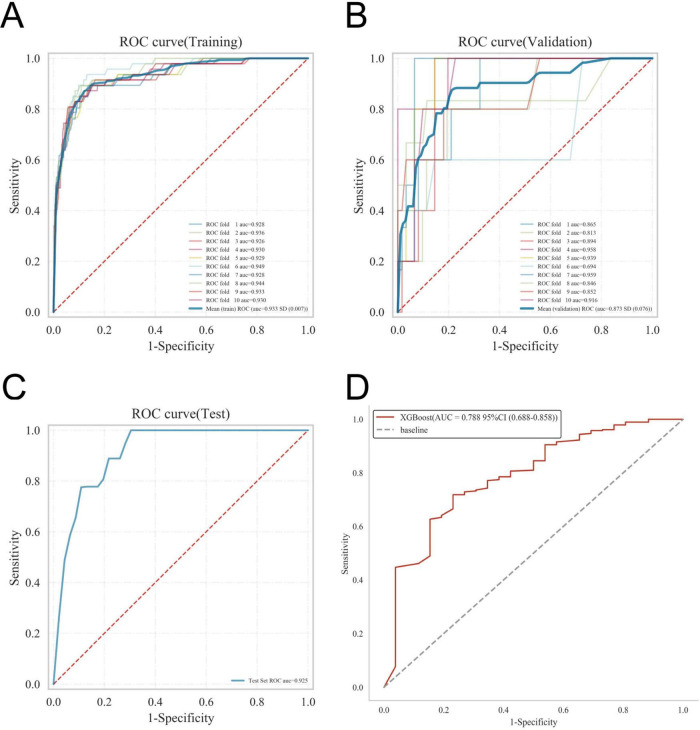
Internal validation of the XGBoost model. **(A)** ROC curve of the XGBoost model for the training set. **(B)** ROC curve of the XGBoost model for the validation set. **(C)** ROC curve of the XGBoost model for the test set. **(D)** External validation of the XGBoost model.

The ROC curve analysis demonstrated that the XGBoost model achieved the highest performance among the four models, with an AUC value of 0.939 in the training set and 0.876 in the validation set, outperforming the other three models (Table 3). The calibration curves of all models closely followed the ideal 45 diagonal, indicating a strong alignment between the predicted probabilities and the actual outcomes. Additionally, the DCA curves revealed that all four models provided a net clinical benefit when compared to both full treatment and no treatment scenarios (Figures 3A–D). The study evaluated the generalization ability of the four models using *k*-fold cross-validation. A total of 118 cases (15.00%) from the internal validation set were selected as the validation set, while the remaining samples were used as the training set. The models were subjected to 10-fold cross-validation. For the XGBoost algorithm, the AUC value in the validation set was 0.8735 ± 0.0764, and the AUC in the test set was 0.9247, with an overall accuracy of 0.8908 (Figures 4A–C). This underscores the exceptional discriminative power and robust generalizability of the XGBoost model, rendering it the most suitable choice for the present study. In contrast, the RF algorithm demonstrated an AUC value of 0.8321 ± 0.0415 in the validation set, with a corresponding AUC of 0.8566 in the test set, yielding an accuracy of 0.8113. The SVM algorithm exhibited an AUC of 0.8061 ± 0.0647 in the validation set and 0.7324 in the test set, with an accuracy of 0.8143. The KNN algorithm, on the other hand, recorded an AUC of 0.7852 ± 0.0654 in the validation set, and 0.7054 in the test set, achieving an accuracy of 0.8864. Following a thorough comparative analysis, the XGBoost algorithm was selected as the foundation for the predictive model in this investigation.

**TABLE 3:** Evaluation of the performance of the four models in the internal validation set.

		AUC (95% CI)	Accuracy (95% CI)	Sensitivity (95% CI)	Specificity (95% CI)	F1 score (95% CI)
KNN	Training set	0.936 (0.897–0.975)	0.949 (0.949–0.949)	0.417 (0.383–0.451)	0.991 (0.989–0.992)	0.542 (0.517–0.568)
	Validation set	0.735 (0.604–0.866)	0.899 (0.886–0.911)	0.129 (0.121–0.137)	0.982 (0.962–1.003)	0.201 (0.181–0.220)
XGBoost	Training set	0.939 (0.901–0.978)	0.892 (0.876–0.907)	0.868 (0.865–0.871)	0.894 (0.877–0.910)	0.538 (0.496–0.580)
	Validation set	0.876 (0.796–0.957)	0.829 (0.817–0.842)	0.706 (0.498–0.915)	0.842 (0.807–0.878)	0.445 (0.374–0.516)
RF	Training set	0.887 (0.834–0.941)	0.862 (0.839–0.886)	0.78 (0.732–0.828)	0.869 (0.840–0.898)	0.452 (0.430–0.473)
	Validation set	0.852 (0.758–0.946)	0.813 (0.770–0.857)	0.74 (0.597–0.883)	0.821 (0.788–0.854)	0.439 (0.318–0.559)
SVM	Training set	0.932 (0.891–0.973)	0.924 (0.923–0.926)	0.791 (0.765–0.817)	0.935 (0.935–0.935)	0.602 (0.584–0.620)
	Validation set	0.858 (0.770–0.946)	0.864 (0.845–0.883)	0.677 (0.657–0.698)	0.884 (0.863–0.906)	0.494 (0.484–0.505)

CI, confidence interval; KNN, *k*-nearest neighbor; XGBoost, extreme gradient boosting; RF, random forest; SVM, support vector machine; AUC, area under the curve.

### Model external validation

The disease prediction model demonstrated exceptional accuracy, as reflected by an AUC value of 0.788 in the external validation set (Figure 4D).

### Model explanation

**FIGURE 5:**
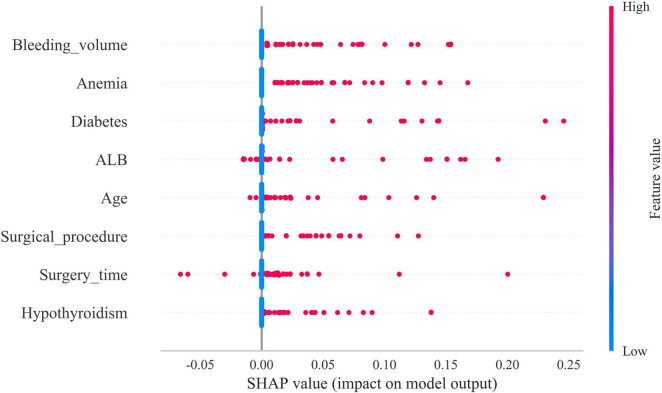
Shapley Additive Explanations (SHAP) summary plot. Risk factors are arranged along the *y*-axis based on their importance, which is given by the mean of their absolute Shapley values. The higher the risk factor is positioned in the plot, the more important it is for the model.

The SHAP summary plot revealed that multiple risk factors contribute to the development of gastroparesis following CME, with intraoperative bleeding exceeding 100 ml, a history of anemia, diabetes mellitus, hypoproteinemia, age ≥65, open surgery, operative duration ≥270 min, and a history of hypothyroidism emerging as the most influential determinants (Figure 5).

**FIGURE 6:**
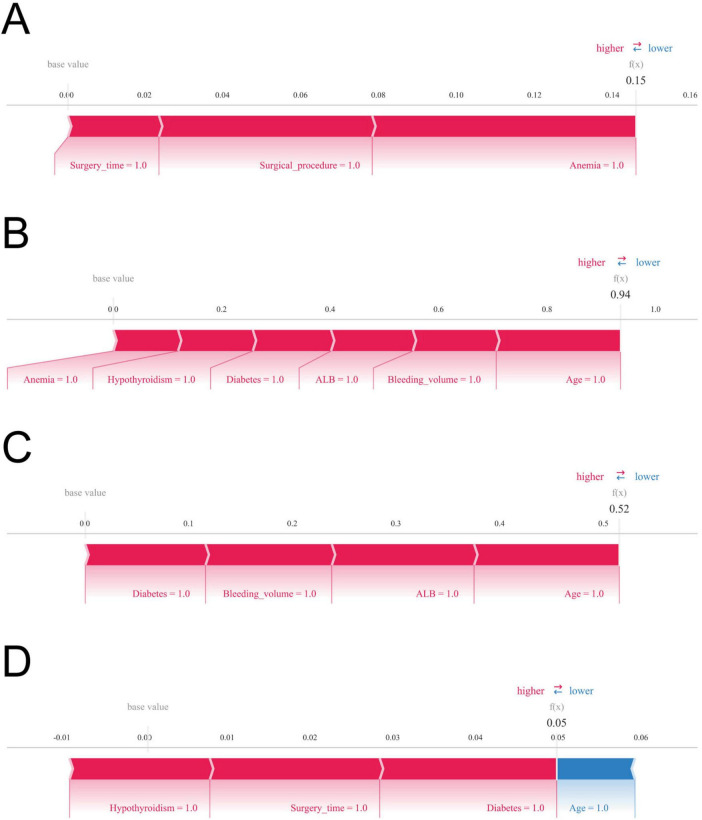
Shapley Additive Explanations (SHAP) force plot. The contributing variables are arranged in the horizontal line, sorted by the absolute value of their impact. Blue represents features that have a negative effect on disease prediction, with a decrease in SHAP values; red represents features that have a positive effect on disease prediction, with an increase in SHAP values. **(A)** Predictive analysis of patient 1. **(B)** Predictive analysis of patient 2. **(C)** Predictive analysis of patient 3. **(D)** Predictive analysis of patient 4.

The SHAP force diagram illustrates the predictive analysis of the study model for four colon cancer patients with gastroparesis. For patient 1, the model predicted a gastroparesis probability of 0.15, with contributing factors including a history of anemia, open surgery, and an operative duration ≥270 min. For patient 2, the predicted probability was 0.94, influenced by a history of anemia, intraoperative bleeding ≥100 ml, a history of hypothyroidism, hypoproteinemia, a history of diabetes mellitus, and age ≥65. In patient 3, the predicted probability of gastroparesis was 0.52, with risk factors including a history of diabetes mellitus, intraoperative bleeding ≥100 ml, hypoproteinemia, and age ≥65. Lastly, patient 4 had a predicted probability of 0.05, with contributing factors such as hypoproteinemia, operative time ≥270 min, and a history of diabetes mellitus, while age ≥65 decreased the probability (Figures 6A–D).

## Discussion

In the present study, SHAP analysis was employed to visualize and interpret the model, revealing that advanced age, prolonged surgical duration, excessive intraoperative bleeding, surgical approach, hypoproteinemia, anemia, and a history of diabetes mellitus and hypothyroidism are significant risk factors for gastroparesis following CME. Traditionally, imaging tests such as CT, MRI, and gastrointestinal fluoroscopy are utilized to diagnose postoperative gastroparesis. However, these diagnostic tools are not only costly but may also subject patients to discomfort and additional medical risks. By leveraging the predictive model developed in this study, clinicians can assess the risk of gastroparesis in advance, based on clinical data and patient-specific characteristics, thus minimizing the need for unnecessary imaging procedures. This approach enhances diagnostic and treatment efficiency while reducing the number of tests required during the diagnostic process. The machine learning model constructed here offers a precise method for identifying patients at high risk of developing gastroparesis after surgery, enabling early detection and the provision of personalized care, ultimately improving the effectiveness of clinical intervention.

The present study sought to assess the performance of four machine learning algorithms in developing risk prediction models. The XGBoost algorithm exhibited remarkable accuracy, distinguished by its efficiency, flexibility, and adaptability, making it an optimal choice for this analysis (15). In contrast to the RF algorithm, the XGBoost algorithm adopts a gradient boosting integration approach that emphasizes difficult-to-classify samples, thereby enhancing generalization performance and ensuring greater stability of the model (16). The SVM and KNN algorithms also exhibited high accuracy and effectively mitigated overfitting issues. However, in the context of managing multiple features and large datasets, the XGBoost algorithm utilizes both L1 and L2 regularization techniques to address overfitting more effectively. Moreover, XGBoost is capable of automatically handling missing values and offers valuable insights into the contribution of each feature to the prediction, enhancing its interpretability. This characteristic renders it particularly advantageous for complex, multidimensional studies. As a result, following a thorough comparison of the four machine learning algorithms, the XGBoost algorithm was selected to develop the predictive model for the occurrence of gastroparesis after CME.

Extreme Gradient Boosting is an ensemble method based on decision trees, specifically utilizing Gradient Boosting Trees. While it excels in numerous tasks, its inherent model complexity can still present the risk of overfitting. One key parameter influencing this complexity is max_depth, which controls the depth of the decision tree. If max_depth is set too high, the model becomes capable of capturing intricate data patterns, including noise and outliers present in the training set. This can lead to overfitting, adversely impacting the model’s performance on unseen data. Additionally, XGBoost operates through integrated learning, constructing multiple trees. If the number of trees is excessive, the individual fitting capacity of each tree increases, which may result in overfitting of the training data by the ensemble model. Furthermore, the learning rate plays a crucial role in determining the contribution of each tree to the overall model. A smaller learning rate causes the model to learn at a slower pace with each iteration, necessitating a larger number of trees to gradually fit the data. While this can enhance model stability, an overly large number of trees can cause the model to overfit the training set. To mitigate the risk of overfitting, we controlled max_depth to limit the tree’s depth, thereby preventing the model from fitting noise or irrelevant data. Additionally, reducing the number of trees (n_estimators) helps avoid overfitting. XGBoost also offers regularization parameters, such as gamma, lambda, and alpha, which can be adjusted to manage model complexity and further prevent overfitting. Finally, we adjusted the learning_rate and n_estimators to balance stability and generalization. Lower learning rates typically require more trees to fit the data, but they contribute to improved generalization, avoiding excessive overfitting. Through these practices, we effectively enhanced the stability and generalization ability of the model, enabling it to perform optimally in practical applications.

Studies (17, 18) have highlighted the efficacy of machine learning algorithms in clinical diagnosis and prognosis, revealing their superior ability to predict adverse outcomes in disease progression compared to traditional diagnostic methods. As individuals age, the digestive system undergoes a range of changes, including a decline in the secretion of digestive enzymes and a reduction in the peristaltic capacity of the smooth muscles within the gastrointestinal tract (19). Moreover, elderly patients often suffer from chronic cardiovascular and respiratory conditions that can affect the nerves and muscles controlling the digestive system, leading to delayed gastric emptying. With a diminished capacity for compensatory function, older individuals exhibit a reduced ability to withstand surgical stress, further exacerbating disruptions in gastrointestinal motility. As a result, postoperative gastroparesis in the elderly arises from a convergence of multiple factors. A study by Meng et al. (20), which included 563 oncology patients, revealed a strong correlation between advanced age and the development of postoperative gastroparesis following abdominal surgery, thereby reinforcing the conclusions of the present study.

The present study corroborates prior research by identifying surgery as a significant determinant of postoperative complications (21, 22). The surgical procedure itself can cause damage to the stomach’s nerves and muscles, impairing both contraction and emptying functions. Traditional open radical colon cancer surgery, which involves an incision in the lower left abdomen, is associated with extended operation times, extensive resections, considerable postoperative pain, and slower patient recovery. In contrast, laparoscopic surgery offers superior precision and convenience, allowing the surgeon to intuitively evaluate lesion size and surrounding tissues, while performing intricate procedures such as tissue dissection and intestinal anastomosis. This approach minimizes damage to healthy gastrointestinal tissues, promoting faster recovery of postoperative gastrointestinal motility. Numerous studies have shown that patients undergoing laparoscopic surgery experience more rapid gastrointestinal recovery and a lower incidence of postoperative complications, including those related to gastric and intestinal function (23, 24). Consequently, we assert that the type of surgery is a pivotal factor in the onset of postoperative gastroparesis. This study further identified prolonged operative duration and increased intraoperative bleeding as significant contributors to the development of gastroparesis following surgery. These factors are often associated with the complexity of the surgical procedure and the heightened risk of inadvertent injury to gastric omental vessels and lymph nodes, which can trigger an exacerbated inflammatory response and disrupt the normal motility of the gastrointestinal tract (25). Furthermore, excessive intraoperative bleeding can compromise hemodynamics within the gastric mucosa and other intestinal segments, thereby delaying the recovery of gastrointestinal tissues. Prolonged surgical durations often require increased administration of anesthetic agents, which inhibit sympathetic constrictor nerve fibers, causing vascular smooth muscle relaxation and a reduction in blood pressure. This drop in blood pressure can precipitate severe complications, including gastroparesis. Additionally, anesthetic drugs may interfere with vagal nerve function, leading to persistent spasm of the pyloric sphincter and impairing the efficiency of gastric emptying (26). Therefore, it is imperative for the surgical team to meticulously plan the procedure in advance and collaborate seamlessly during the operation to enhance surgical efficiency, reduce operative time, and minimize the risk of postoperative gastroparesis.

Furthermore, this study aimed to illuminate the underlying rationale behind the postoperative gastroparesis prediction model using four distinct samples. In the disease prediction analysis of the second sample, the patient’s nutritional status emerged as a pivotal predictor. Plasma albumin, despite its modest molecular weight, plays an indispensable role in maintaining both plasma and tissue fluid osmolality. Hypoalbuminemia results in reduced plasma colloid osmotic pressure, heightening the risk of intestinal wall edema and disrupting gastrointestinal motility. Moreover, patients with hypoalbuminemia exhibit diminished numbers and activity of antibody synthase, thereby increasing their vulnerability to complications such as postoperative infections and anastomotic leakage, which further hinder gastrointestinal recovery. These findings emphasize the critical need for clinicians to carefully assess preoperative albumin levels and promptly manage postoperative complications. Enhancing parenteral nutrition for patients with hypoproteinemia, coupled with the supplementation of a high-protein diet when feasible, may help alleviate the risk of hypoalbuminemia and reduce the likelihood of postoperative gastroparesis (27). Similarly, patients with anemia are more susceptible to adverse outcomes, including postoperative gastroparesis. Right hemicolectomy patients frequently experience tumor infiltration that disrupts mucosal and submucosal vessels in the colon lining, contributing to malnutrition-related conditions such as iron deficiency anemia. Hemoglobin, vital for oxygen transport in the bloodstream, is indispensable for maintaining adequate oxygen supply to bodily tissues. Anemia leads to tissue hypoxia, a result of insufficient hemoglobin, which severely impairs the normal functioning of gastrointestinal smooth muscles. Additionally, the small perigastric vessels play a crucial role in nourishing the perigastric vagus nerve. Al-Saffar et al. (28) demonstrated that preoperative hemoglobin levels serve as an independent risk factor for delayed gastric emptying. The incidence of gastroparesis is markedly higher in anemic patients compared to their non-anemic counterparts, underscoring the imperative for vigilant monitoring of gastrointestinal function in anemic individuals and the prompt initiation of preventive strategies to mitigate gastrointestinal complications.

The present investigation further reveals that the underlying condition significantly influences the onset of postoperative gastroparesis. Diabetic patients often manifest neuropathic alterations that impair both their autonomic and visceral nerves, thereby hindering the motility of the smooth muscles within the gastrointestinal tract (29). Farrugia (30) demonstrated that a reduction in Cajal mesenchymal cells, key regulators of gastrointestinal motility, constitutes a critical mechanism for delayed gastric emptying in an animal model, thereby providing further validation for the findings of the present study. Furthermore, we posit that fluctuations in blood glucose levels resulting from perioperative fasting serve as a significant trigger for the onset of gastroparesis in these patients. Postoperatively, many of these patients develop insulin resistance, which hampers the secretion and release of gastrin and disrupts the function of the autonomic nervous system, thereby impairing gastric emptying (31). The rate of gastric emptying was found to be closely correlated with diabetes in a comprehensive, multicenter study conducted over 30 years by clinical researchers worldwide (31, 32). Similarly, individuals with hypothyroidism exhibit comparable effects. Thyroxine plays a crucial role in sustaining the body’s physiological functions, particularly in motor function. However, clinicians often focus primarily on limb movements in hypothyroid patients, neglecting the activity of glandular organs. Pathologists who conducted biopsies of gastric tissues from hypothyroid patients observed edema and thickening of gastric mucosal cells, accompanied by surrounding inflammatory cell infiltration (33). Building on this, Ghoshal et al. (34) conducted a clinical study involving 60 hypothyroid patients and found a strong correlation between reduced thyroxine levels and impaired gastrointestinal motility. It is posited that enhanced vagal function in hypothyroid patients results in hyperexcitability, disrupting the normal coordination and rhythmicity of the gastrointestinal smooth muscles. Experimental findings by Khraisha et al. (35) in patients with chronic atrophic gastritis and gastroparesis further corroborate the therapeutic potential of thyroxine in addressing gastric motility disorders.

The present study thoroughly assessed the model’s performance across differentiation, calibration, and clinical utility. However, several limitations warrant consideration. The predictive models were primarily based on clinical indicators and laboratory data, without incorporating imaging data such as CT, MRI, or ultrasound scans. While we acknowledge the significant role of imaging data in enhancing prediction accuracy and precision, the lack of imaging data in our analysis was due to constraints in available data sources. Future studies that integrate both imaging and clinical data could potentially further refine the model’s predictive capability. Additionally, while machine learning algorithms offer superior accuracy, they tend to be more complex and less interpretable. The computational and decision-making processes of these models operate in a “black box,” which diminishes the intuitiveness and transparency associated with more traditional approaches, such as logistic regression modeling (36). This study was retrospective in nature, relying on historical patient data or electronic health records. Due to the absence of real-time interventions and standardized treatment protocols, retrospective data may suffer from incomplete documentation or inherent biases, potentially affecting the reliability of the findings. Furthermore, the study sample was not randomized, which introduces selection bias and may limit the generalizability of the results. Additionally, because the data were sourced from a specific hospital or region, the patient population characteristics—such as age, gender, and underlying conditions—may differ from those in other regions or populations. This distributional bias could undermine the external validity of the model, particularly its applicability to other regions or patient groups. Variations in medical resources, treatment standards, and genetic backgrounds across different regions could also lead to differing disease presentations and postoperative recovery, further impacting the model’s broader applicability. Moreover, we acknowledge that certain potential confounders, such as anastomotic leakage leading to severe infections and impaired nutrient absorption, may exacerbate the risk of gastroparesis. However, these variables were not included due to data limitations and study design constraints. In future research, we plan to conduct a multicenter, prospective study to enhance the external validation of our model, ensuring its predictive accuracy across diverse clinical settings.

## Conclusion

Identifying high-risk patients for gastroparesis after CME enables the implementation of timely interventions to enhance patient outcomes. The predictive model exhibited exceptional accuracy and strong clinical utility, offering surgeons a valuable tool for early diagnosis and proactive management. The analysis highlighted that the development of postoperative gastroparesis in colon cancer patients is strongly associated with advanced age, extended operative time, substantial intraoperative bleeding, surgical approach, hypoproteinemia, anemia, and preexisting conditions such as diabetes mellitus and hypothyroidism.

## Data Availability

The datasets presented in this study can be found in online repositories. The names of the repository/repositories and accession number(s) can be found in this article/Supplementary material.

## Supplementary material

Supplementary Table 1 Raw data.
